# Value of radiological depth of invasion in non-pT4 Oral tongue squamous cell carcinoma: implication for preoperative MR T-staging

**DOI:** 10.1007/s00330-024-10598-7

**Published:** 2024-02-03

**Authors:** Wenjie Huang, Yu Zhang, Gui Fu, Manqian Huang, Guangfeng Luo, Hui Xie, Zhiying Liang, Di Cao, Shuqi Li, Chao Luo, Haojiang Li, Jiexin Gao, Rongcheng Nie, Guangying Ruan, Hao Li, Lizhi Liu

**Affiliations:** 1 State Key Laboratory of Oncology in South China, Guangdong Key Laboratory of Nasopharyngeal Carcinoma Diagnosis and Therapy, Guangdong Provincial Clinical Research Center for Cancer, Sun Yat-Sen University Cancer Center, Guangzhou, 510060 China; 2 Department of Radiology, Sun Yat-Sen University Cancer Center, Guangzhou, China; 3 Department of Pathology, Sun Yat-Sen University Cancer Center, Guangzhou, China; 4 Department of Oral & Maxillofacial Surgery, Sun Yat-Sen University Cancer Center, Guangzhou, China; 5 Department of Mathematics, Faculty of Science and Technology, University of Macau, Macao, China

**Keywords:** Squamous cell carcinoma, Tongue cancer, Magnetic resonance imaging, Depth of invasion, Tumor thickness

## Abstract

**Objective:**

The prognostic stratification for oral tongue squamous cell carcinoma (OTSCC) is heavily based on postoperative pathological depth of invasion (pDOI). This study aims to propose a preoperative MR T-staging system based on tumor size for non-pT4 OTSCC.

**Methods:**

Retrospectively, 280 patients with biopsy-confirmed, non-metastatic, pT1-3 OTSCC, treated between January 2010 and December 2017, were evaluated. Multiple MR sequences, including axial T2-weighted imaging (WI), unenhanced T1WI, and axial, fat-suppressed coronal, and sagittal contrast-enhanced (CE) T1WI, were utilized to measure radiological depth of invasion (rDOI), tumor thickness, and largest diameter. Intra-class correlation (ICC) and univariate and multivariate analyses were used to evaluate measurement reproducibility, and factors’ significance, respectively. Cutoff values were established using an exhaustive method.

**Results:**

Intra-observer (ICC = 0.81–0.94) and inter-observer (ICC = 0.79–0.90) reliability were excellent for rDOI measurements, and all measurements were significantly associated with overall survival (OS) (all *p* < .001). Measuring the rDOI on axial CE-T1WI with cutoffs of 8 mm and 12 mm yielded an optimal MR T-staging system for rT1-3 disease (5-year OS of rT1 vs rT2 vs rT3: 94.0% vs 72.8% vs 57.5%). Using multivariate analyses, the proposed T-staging exhibited increasingly worse OS (hazard ratio of rT2 and rT3 versus rT1, 3.56 [1.35–9.6], *p* = .011; 4.33 [1.59–11.74], *p* = .004; respectively), which outperformed pathological T-staging based on nonoverlapping Kaplan–Meier curves and improved C-index (0.682 vs. 0.639, *p* < .001).

**Conclusions:**

rDOI is a critical predictor of OTSCC mortality and facilitates preoperative prognostic stratification, which should be considered in future oral subsite MR T-staging.

**Clinical relevance statement:**

Utilizing axial CE-T1WI, an MR T-staging system for non-pT4 OTSCC was developed by employing rDOI measurement with optimal thresholds of 8 mm and 12 mm, which is comparable with pathological staging and merits consideration in future preoperative oral subsite planning.

**Key Points:**

*• Tumor morphology, measuring sequences, and observers could impact MR-derived measurements and compromise the consistency with histology.*

*• MR-derived measurements, including radiological depth of invasion (rDOI), tumor thickness, and largest diameter, have a prognostic impact on OS (all p < .001).*

*• rDOI with cutoffs of 8 mm and 12 mm on axial CE-T1WI is an optimal predictor of OS and could facilitate risk stratification in non-pT4 OTSCC disease.*

**Supplementary Information:**

The online version contains supplementary material available at 10.1007/s00330-024-10598-7.

## Introduction

Depth of invasion (DOI) and tumor thickness (TT) are related but distinct pathological features that have significant roles in the prognosis of oral cavity cancers [1, 2]. Pathological DOI (pDOI) is defined as the perpendicular distance from the level of the epithelial basement membrane to the deepest point of tumor infiltration, irrespective of ulcerative or exophytic growth pattern [3]. This differs from pathological TT (pTT), which represents the thickness of the tumor and contains the invasive and exogenous portion [4]. The greater importance of the former has resulted in its incorporation into the 8th American Joint Committee on Cancer (AJCC) staging system for describing pT1-pT3 primary disease (non-pT4) using 5 mm and 10 mm as cutoff values [5], with tumors showing a pDOI > 10 mm being classified as advanced and exhibiting a 5-year disease-specific mortality close to 40% [3]. As such, more importance is placed on preoperative alignment to histology and thus on accumulating radiological DOI (rDOI)/tumor thickness (rTT)–correlated measurements based on multiple imaging modalities [6–9]. However, despite their high consistency with the pDOI, these MR measurements are derived from small datasets, classically categorize whole-staged patients into two risk subgroups, and lack comparison across different measurements of tumor size, particularly those displaying divergent agreement, which makes preoperative clinical staging challenging. Determining which MR sequence, dimension, and tumor size to measure, with priority given to survival estimation, is crucial for improving the accuracy of prognostic assessments in oral cancer.

Staging systems for head and neck cancers have now begun to reflect the clinical characteristics of distinct anatomical locations more accurately. For example, the preoperative primary staging of nasopharyngeal carcinomas includes detailing radiological features for the quantification of tumor extension [10]. However, although oral tongue squamous cell carcinoma (OTSCC) accounts for more than half of oral cancers and exhibits more aggressive clinical behavior [11, 12], its risk stratification and treatment planning still follow the general oral staging system. This includes all subregions and has suboptimal prognostic performance [13, 14]. Hence, a preoperative primary radiological staging system specific to OTSCC is needed. MR is recommended as the optimal examination tool for assessing the extent of tumor infiltration [15]. Developing a practical, preoperative, MR-based measurement of tumor size is of great importance for the evaluation of the specific subsite of the head and neck and its risk stratification. This study aimed to propose a preoperative MR T-staging system based on the tumor size of non-pT4 OTSCC.

## Materials and methods

### Study design and patients

**Fig. 1:**
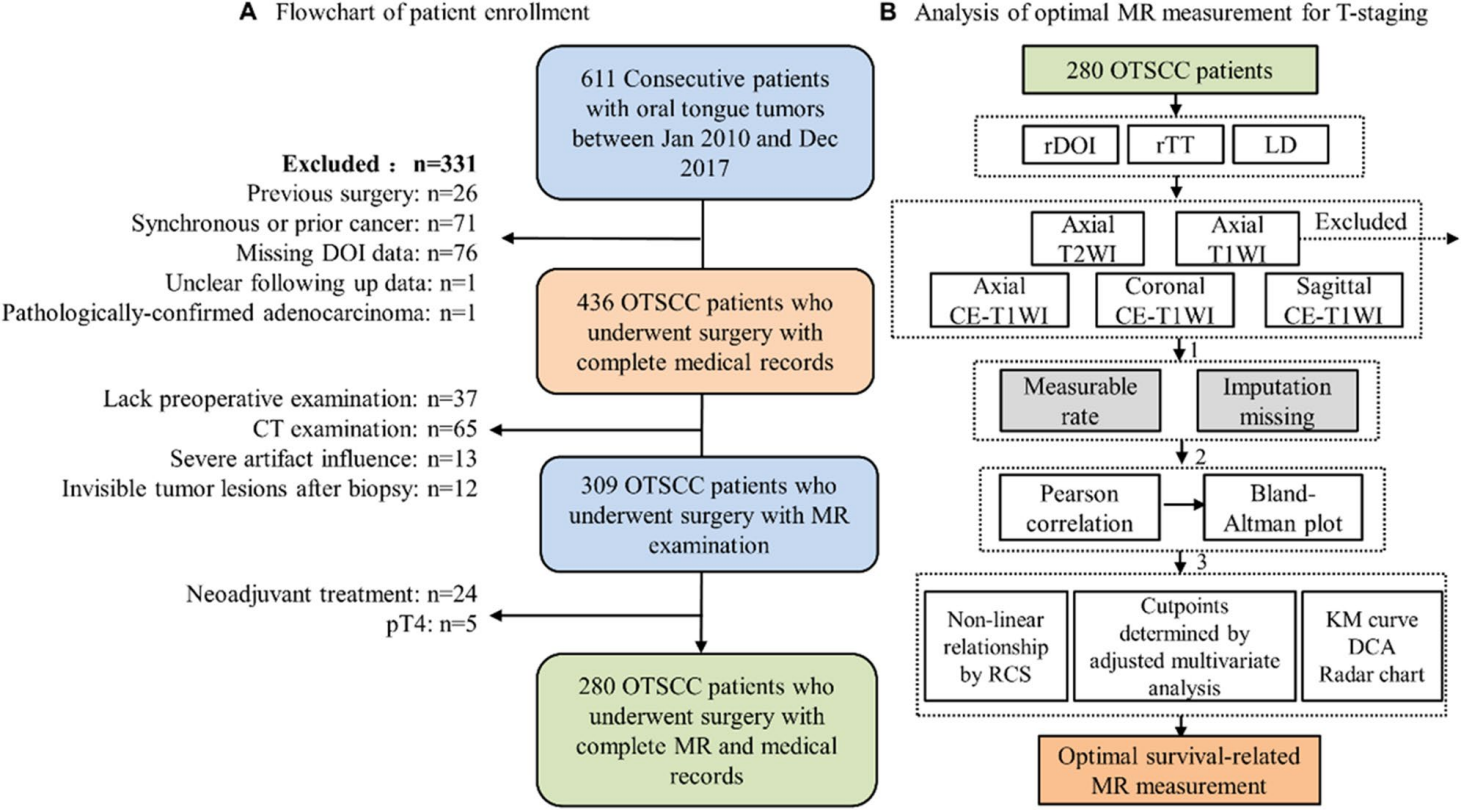
Study flowchart. Flowchart depicting (**A**) inclusion and exclusion criteria of the patients and (**B**) statistical steps for determining the optimal MR measurement through multiple MR sequences for non-pT4 OTSCC primary tumor staging. Abbreviations: KM, Kaplan–Meier; DCA, decision curve analysis; RCS, restricted cubic spline; CE, contrast-enhanced; OTSCC, oral tongue squamous cell carcinoma; rDOI, radiological depth of invasion; rTT, radiological tumor thickness; LD, longest diameter

This retrospective study was approved by the institutional review board and followed the tenets of the Declaration of Helsinki. The requirement for informed consent was waived owing to the retrospective nature of the study. In total, 611 consecutive patients with oral tongue tumors were enrolled between January 2010 and December 2017. After excluding 331 patients according to the exclusion criteria, 280 patients with surgically resected pT1-3 OTSCC who underwent MR imaging of the head and neck as part of initial staging before surgery were included (Fig. 1A).

### Imaging protocol

All MR examinations were performed using a 1.5-T unit (Signa CV/i, General Electric Healthcare) or a 3.0-T system (Magnetom Tim Trio, Siemens) with a head-and-neck combined coil. For all MR examinations, patients were placed in the supine position with mandibular adduction. Pretreatment training for avoiding swallowing and possible removal of active dentures at the side of lesions was provided to ensure that each patient completed approximately 20–25 min of examination. The unenhanced image sequences included fast-spin echo T1-weighted imaging (WI) (repetition time = 540 ms, echo time = 11.8 ms) in the axial, coronal, and sagittal planes and fast-spin echo T2WI (repetition time = 4000 ms, echo time = 99 ms) and diffusion-weighted imaging in the axial planes. After intravenous injection of 0.1 mmol/kg contrast agent (Gd-DTPA, Magnevist, Schering), the axial and sagittal T1WI and coronal T1WI fat-suppressed sequences were recorded. The section thicknesses were 4 mm and 3 mm for the axial and sagittal planes, respectively, both with section gaps of 1 mm, and 2 mm for the coronal plane with section gaps of 0.5 mm.

### MR imaging measurement

**Fig. 2:**
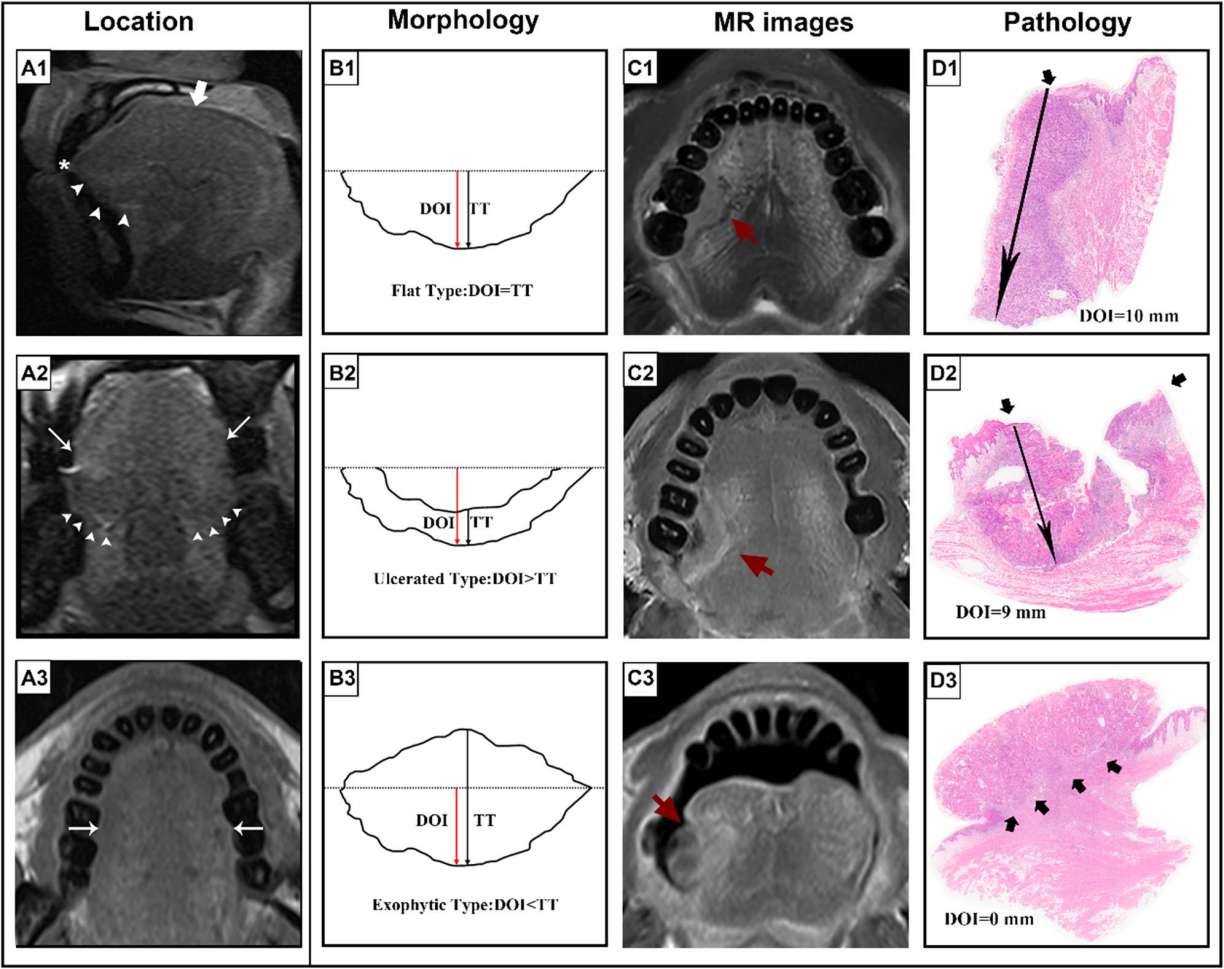
Diagram of tumor location, morphology, and corresponding MR images and pathology. For tumor location (**A1**–**A3**), we defined tip (asterisk), dorsum (thick arrow), ventral (arrowhead), and lateral (thin arrow) parts on sagittal, coronal, and axial views of MR according to anatomic criteria, respectively. For tumor morphology (**B1**–**B3**), the diagram depicts the measurement of rTT, rDOI, and LD in flat, ulcerative, and exophytic types, respectively. The red line refers to the measurement of rDOI from the vertical distance between the simulated normal mucosal junction to the deepest point of tumor infiltration. The black line refers to measurement of rTT by drawing a vertical line from the tumor surface to the deepest point of infiltration. In the following columns (**C1**–**C3**), the short red arrow refers to the lesions of corresponding morphology on the MR imaging, and the long black arrow refers to the pDOI on histological sections (**D1**–**D3**). Specifically for the histological sections, the short black arrow refers to the level of epithelial basement membrane but no DOI was obtained due to the AJCC criteria (**D3**). Abbreviations: DOI, depth of invasion; LD, longest diameter; rDOI, radiological depth of invasion; rTT, radiological tumor thickness; AJCC, American Joint Committee on Cancer

**Fig. 3:**
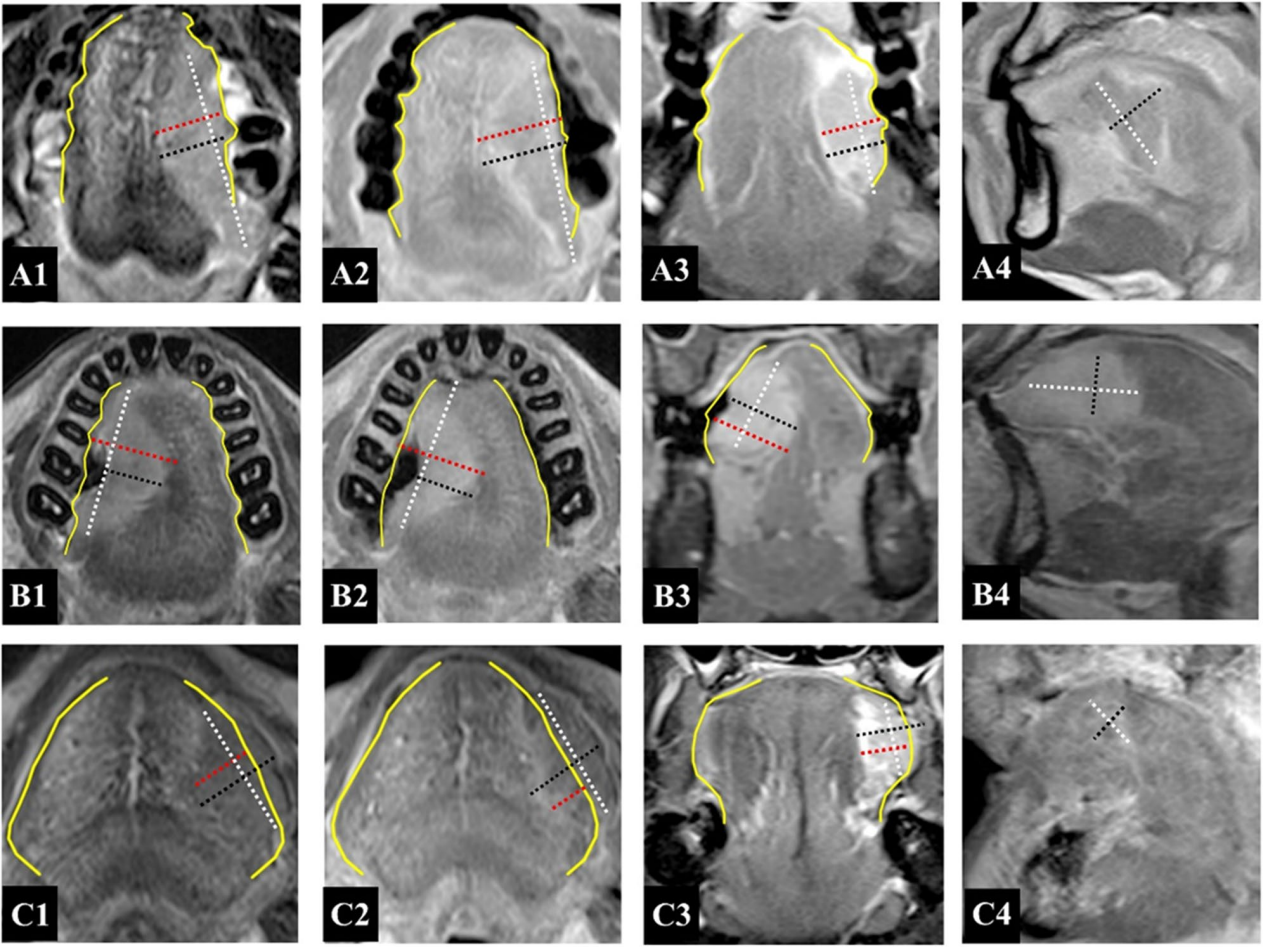
Diagram of tumor measurement of different morphologies. Upper (**A1**–**A4**), middle (**B1**–**B4**), and lower (**C1**–**C4**) row from left to right using black, red, and white dashed line representing measurement of rTT, rDOI, and LD in flat, ulcerative, and exophytic case on axial T2WI, and axial, fat-suppressed coronal, and sagittal CE-T1WI sequence, respectively. Yellow line represents the simulated normal mucosal junction. Abbreviations: rTT, radiological tumor thickness; rDOI, radiological depth of invasion; LD, largest diameter

Drawing a vertical line from the horizon connecting the adjacent normal basement membrane according to the 8th AJCC pDOI criteria [5] would result in an underestimation of depth due to the naturally curved surface of the tongue. Thus, we measured the rDOI from the vertical distance between the simulated normal mucosal junction and tumor invasion front [7]. The contralateral normal side was used as the reference for reconstructing the simulated curved mucosal surface. We performed MR measurements of the rTT on the axial and coronal views mediolaterally and on the sagittal view in the supero-inferior direction following the pTT criteria of the 7th AJCC [16], where a vertical line is drawn from the tumor surface to the deepest point of infiltration. The longest diameter (LD) was measured perpendicular to the rTT. We measured each tumor size on consecutive sections and obtained the largest value of the corresponding measurement in millimeters. Measuring all size indicators in all specified sequences required 3–5 min per patient for an experienced radiologist. Figure 2 illustrates a diagram of the rTT and rDOI for different morphologies and the corresponding MR and histological sections. There are no landmarks on the mobile tongue that would objectively discriminate between the ventral and dorsum parts based on radiology. However, there are clear and distinct regions based on the clinical visual examination. Thus, we defined tip, dorsum, ventral, and lateral (blade) parts on sagittal, coronal, and axial views of MR imaging according to anatomic criteria, respectively. The details of measurement of different morphologies are illustrated in Fig. 3.

To assess the effect of inter- and intra-observer variability, 10% (28/280) of the patients were randomly selected and measurements were performed again in these patients after 3 months by the same radiologist (H.W.J.) and a senior radiologist (F.Q.) with 6 years of experience in head and neck diagnosis. Cases of disagreement between the rDOI and pDOI > 10 mm were verified by a more senior radiologist (L.L.Z.) and a senior pathologist (Z.Y.), both with > 20 years of experience in head and neck diagnosis.

### Follow-up and endpoint

All patients were followed up every 1–3 months during the first year and every 3–12 months thereafter. Follow-up until December 2022 was conducted via telephone or outpatient documents. The main clinical endpoint in this study was overall survival (OS), defined as the period from the date of surgery to the date of death from any cause or the last follow-up.

### Statistical analyses

The missing rates for all measurements are summarized in eTable 1. Given that the data were non-randomly missing and the individual’s other sequences could complement the missing, we imputed the missing values using a fully conditional specification implemented by multivariable imputation to reduce the risk of bias [17]. Categorical variables are described using frequency rates and percentages, and continuous variables are expressed as mean ± standard deviation or median ± range. Pearson’s correlation coefficient and Bland–Altman analysis were used to assess the agreement between each MR measurement and the pDOI. Agreements > 0.8, 0.6–0.8, 0.4–0.6, and < 0.4 were considered to indicate excellent, good, moderate, and poor concordance, respectively. Interclass correlation coefficients (ICCs) were calculated to assess the intra- and inter-observer agreement. Measurements with ICC > 0.75 were considered to indicate high reproducibility. Kaplan–Meier analysis with log-rank test was used to compare survival rates, while the likelihood of survival outcomes of hazard ratios (HRs) and their 95% confidence intervals (CIs) were assessed using the multivariate Cox proportional hazards model.

Confounding factors were primarily selected from 13 clinicopathological variables for further adjusted multivariate Cox regression before determining the optimal cutoff point of each measurement for risk stratification. Next, a three-step procedure was designed to determine the optimal MR measurements. The first step used a restricted cubic spline function that allows for the nonlinear association of each MR measurement with OS. The optimal number of cutoffs for categorizing MR-derived measurement was determined based on lowest Akaike information criterion (AIC). Next, an exhaustive algorithm (https://github.com/xh542428798) was used to determine the optimal cutoffs of each MR measurement after adjustment of selected confounding factors [3, 18]. Finally, multiple evaluation criteria were used to select the optimal MR measurement, including the concordance index (C-index), decision curve analysis, and four evaluation criteria defined by Groome et al [19]: hazard consistency, hazard discrimination, sample size balance, and outcome prediction by recursive partitioning analysis (available at http://rpa.renlab.org/). Lower value of hazard discrimination, hazard consistency, sample size balance, and higher outcome prediction indicate better performance of the staging category. The statistical process is summarized in Fig. 1 B. All statistical analyses were performed using the R package (Version 4.2.2; mice, rms, survival, SiZer, party libraries). Statistical significance was set at α = 0.05.

## Results

### Patient characteristics

The mean age of the patients was 53 ± 13 years, and 184 patients were male. The lateral border was the most common tumor location (90.4%), followed by the ventral border (7.5%), dorsum (1.8%), and tip (0.3%). In total, 11 tumors (3.9%) were classified as exophytic, 22 (7.9%) as ulcerative, whereas the remaining 247 patients (88.2%) had neither feature. The baseline patient characteristics are presented in Table 1. The median follow-up time was 60.2 months [95% CI, 54.4–64.8]. In total, 69 patients (24.6%) died during follow-up. The median and 5-year OS was 75.8 months (95% CI, 70.8–81.2) and 73.9%, respectively.

**Table 1:** Characteristics of patients

Variables	*N* = 280	OS		
		5-year (%)	HR [95% CI]	*p* value
Age (year)				0.064
≤ 55	154 (55%)	77.95	**1 (reference)**	
> 55	126 (45%)	69.06	1.56 (0.97–2.51)	
Sex				0.002
Male	184 (65.7%)	67.83	**1 (reference)**	
Female	96 (34.3%)	85.29	0.39 (0.21–0.72)	
Tumor morphology				0.424
Flat type	247 (88.2%)	73.86	**1 (reference)**	
Ulcerative type	22 (7.9%)	68.18	1.39 (0.63–3.03)	
Exophytic type	11 (3.9%)	90	0.39 (0.05–2.79)	
Tumor location^a^				0.781
Lateral	253 (90.4%)	73.15	**1 (reference)**	
Non-Lateral	27 (9.6%)	80.89	0.89 (0.38–2.05)	
Hypertension				0.445
No	235 (83.9%)	73.83	**1 (reference)**	
Yes	45 (16.1%)	74.23	1.26 (0.69–2.31)	
Diabetes				0.255
No	255 (91.1%)	74.31	**1 (reference)**	
Yes	25 (8.9%)	70.18	1.53 (0.73–3.20)	
Smoking				0.01
No	179 (63.9%)	78.47	**1 (reference)**	
Yes	101 (36.1%)	65.71	1.84 (1.15–2.96)	
Alcohol use				0.004
No	225 (80.4%)	77.29	**1 (reference)**	
Yes	55 (19.6%)	59.24	2.09 (1.25–3.49)	
Betel liquid				0.084
No	266 (95%)	74.72	**1 (reference)**	
Yes	14 (5%)	55.53	2.19 (0.88–5.46)	
Family history				0.775
No	265 (94.6%)	73.97	**1 (reference)**	
Yes	15 (5.4%)	71.79	1.16 (0.42–3.18)	
**cTstage**^**b**^				< 0.001
T0	132 (47.1%)	85.01	**1 (reference)**	
T1	110 (39.3%)	66.56	2.33 (1.33–4.09)	
T2	14 (5%)	70.71	2.72 (1.01–7.28)	
T3	24 (8.6%)	49.19	4.34 (2.06–9.13)	
**cNstage**^**b**^				0.088
N0	222 (79.3%)	75.72	**1 (reference)**	
N1	40 (14.3%)	73.61	1.25 (0.63–2.46)	
N2	18 (6.4%)	54.55	2.25 (1.07–4.73)	
**pTstage**^**c**^				< 0.001
T1	94 (33.6%)	90.27	**1 (reference)**	
T2	102 (36.4%)	69.94	3.34 (1.58–7.08)	
T3	84 (30%)	59.84	4.82 (2.30–10.10)	
**pNstage**^**c**^				< 0.001
N0	205 (73.2%)	82.96	**1 (reference)**	
N1	24 (8.6%)	66.11	2.65 (1.26–5.57)	
N2	44 (15.7%)	40.71	5.78 (3.42–9.77)	
N3	7 (2.5%)	31.25	5.04 (1.54–16.53)	
Differentiation^d^				0.164
Non-well differentiated	146 (52.1%)	69.75	**1 (reference)**	
Well differentiated	134 (47.9%)	78.06	0.71 (0.44–0.15)	
Lymphovascular involvement				0.309
No	269 (96.1%)	74.7	**1 (reference)**	
Yes	11 (3.9%)	54.55	1.68 (0.61–4.61)	
Perineural invasion				0.02
No	213 (76.1%)	77.23	**1 (reference)**	
Yes	67 (23.9%)	62.8	1.81 (1.09–3.01)	
Margin status				0.092
Negative	277 (98.9%)	74.57	**1 (reference)**	
Positive	3 (1.1%)	NA	3.15 (0.77–12.87)	
Extranodal extension				0.004
No	272 (97.1%)	74.93	**1 (reference)**	
Yes	8 (2.9%)	27.34	3.92 (1.42–10.80)	
Adjuvant chemotherapy				< 0.001
No	231 (82.5%)	78.34	**1 (reference)**	
Yes	49 (17.5%)	52.31	1.91 (0.95–3.85)	
Adjuvant radiotherapy				0.067
No	255 (91.1%)	75.22	**1 (reference)**	
Yes	25 (8.9%)	59.81	2.75 (1.66–4.57)	

Data are presented as the number (percentages) of patients. *NA* not applicable

^a^Owing to the small sample size of the non-lateral location subgroup, we combined all non-lateral location as one subgroup for better statistical analyses

^b^Referring to preoperative clinical T-staging and N-staging according to the 8th edition of the American Joint Committee on Cancer

^c^Referring to postoperative pathological T-staging and N-staging according to the 8th edition of the American Joint Committee on Cancer

^d^Referring to WHO differentiation grade, which includes well (grade I), moderately (grade II), poorly (grade III), and undifferentiated (grade IV) type. Given that the well-differentiated type accounts for more than half of the whole data set, we characterized the histological grade as well and moderate-poor differentiation subgroups for better statistical analyses

### Correlation between MR-derived measurements and the pDOI

**Fig. 4:**
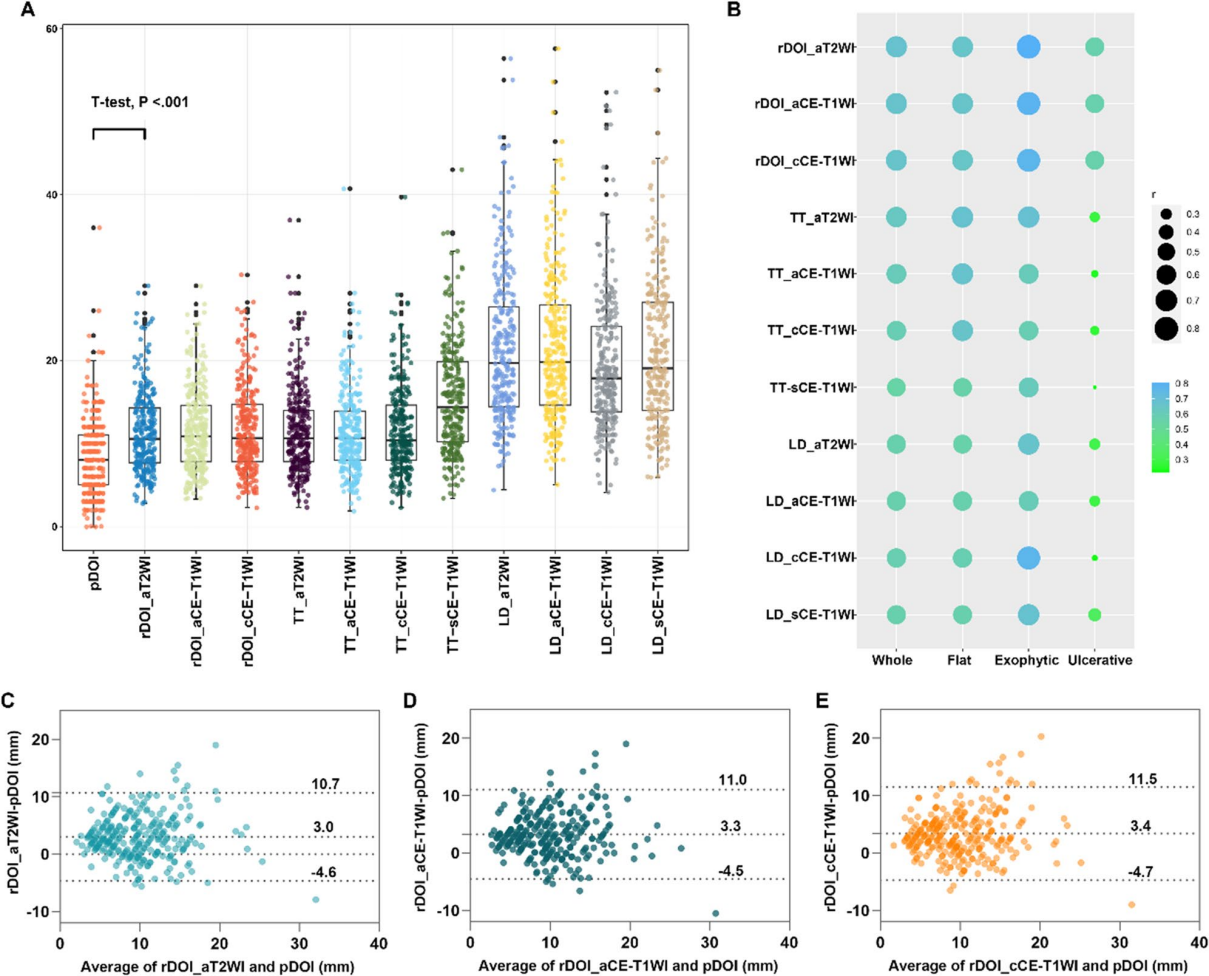
Graphical representation of rDOI, rTT, and LD measurements per sequence and correlation with pDOI. **A** Boxes show the upper and lower quartiles, and horizontal lines within boxes represent median values. Whiskers represent the 95th and 5th percentiles, and the jittering dot indicates the MR measurement for each patient. All measurements were significantly larger than pDOI. **B** The correlation coefficient between pDOI and each MR measurement. **C**–**E** Bland–Altman plots showing the agreement between pDOI and rDOIs. Dashed horizontal lines represent the mean bias, and top and bottom dashed lines represent the upper and lower limits of agreement, respectively. Abbreviations: pDOI, pathological depth of invasion; rDOI, radiological depth of invasion; LD, longest diameter; TT, tumor thickness; aT2WI, axial T2WI; aCE-T1WI, axial contrast-enhanced T1WI; cCE-T1WI, coronal fat-suppressed CE-T1WI; sCE-T1WI, sagittal CE-T1WI

The mean measured rDOI on axial T2WI, axial contrast-enhanced (CE)-T1WI, and coronal fat-suppressed CE-T1WI was 11.4 mm (range, 2.8–29 mm), 11.6 mm (range, 3.3–29 mm), and 11.7 mm (range, 2.3–30.3 mm), respectively, which were significantly larger than the measured pDOI of 8.3 mm (range, 0–36 mm) (Fig. 4A). Similar results were observed for the measurement of rTT and LD on axial T2WI and CE-T1WI, and coronal fat-suppressed and sagittal CE-T1WI (Table 2). Figure 4 B shows the correlation between pDOI and each MR measurement.

**Table 2:** rTT, rDOI, and LD measured on each sequence

Measurement	*N* = 280	OS	
		HR [95% CI]	*p* value
pDOI (mm)	8.3 ± 4.8	1.08 (1.04–1.12)	< 0.001
rTT_aT2WI (mm)	11.4 ± 5.1	1.10 (1.06–1.15)	< 0.001
rTT_aCE-T1WI (mm)	11.4 ± 5	1.12 (1.07–1.16)	< 0.001
rTT_cCE-T1WI (mm)	11.7 ± 5.3	1.10 (1.06–1.14)	< 0.001
rTT_sCE-T1WI (mm)	15.4 ± 6.6	1.09 (1.06–1.13)	< 0.001
rDOI_aT2WI (mm)	11.4 ± 4.9	1.11 (1.07–1.14)	< 0.001
rDOI_aCE-T1WI (mm)	11.6 ± 4.9	1.08 (1.05–1.12)	< 0.001
rDOI_cCE-T1WI (mm)	11.7 ± 5.2	1.06 (1.03–1.09)	< 0.001
LD_aT2WI (mm)	21.4 ± 9	1.06 (1.03–1.08)	< 0.001
LD_aCE-T1WI (mm)	21.6 ± 9	1.05 (1.02–1.07)	< 0.001
LD_cCE-T1WI (mm)	19.4 ± 8.1	1.05 (1.03–1.07)	< 0.001
LD_sCE-T1WI (mm)	20.8 ± 8.7	1.05 (1.03–1.08)	< 0.001
rTTmax* (mm)	16.1 ± 6.6	1.06 (1.03–1.10)	< 0.001
rDOImax* (mm)	12.7 ± 5.1	1.11 (1.07–1.15)	< 0.001
LDmax* (mm)	24.5 ± 9.2	1.05 (1.03–1.08)	< 0.001

Data are presented as the mean ± standard deviation of patients

^*^We have derived the following three variables that mimic the current preoperative T-staging, including LDmax, the largest value of all LD measurements in each patient, and rTTmax and rDOImax, the largest values of all rTT and rDOI measurements in each patient, respectively

*Abbreviation*: *rTT* radiological tumor thickness, *rDOI* radiological depth of invasion, *LD* largest diameter, *pDOI* pathological depth of invasion, *aT2WI* axial T2WI, *aCE-T1WI* axial contrast-enhanced T1WI, *cCE-T1WI* coronal fat-suppressed CE-T1WI, *sCE-T1WI* sagittal CE-T1WI

There was a good positive correlation between the rDOI and pDOI in the entire study sample, with *r* values of 0.67, 0.67, and 0.66 on axial T2WI, axial, and fat-suppressed coronal CE-T1WI, respectively. Furthermore, we observed good positive correlations between the rDOI and pDOI in exophytic (*r* = 0.81, 0.78, and 0.77, respectively) and flat type tumors (*r* = 0.66, 0.66, and 0.65, respectively). However, this correlation was moderate in the ulcerative subgroup (*r* = 0.56, 0.57, and 0.56, respectively). The intra- and inter-observer reliabilities of the rDOI measured on axial T2WI, and axial and fat-suppressed coronal CE-T1WI were excellent (ICC = 0.94, 0.95, and 0.81 for intra-reliability; ICC = 0.9, 0.89, and 0.79 for inter-reliability, respectively). Similar results were observed for the rTT and LD on each sequence (0.63–0.97 and 0.73–0.95, respectively; eTable 2).

### Agreement between MR-derived measurements and the pDOI

The Bland–Altman plot showed a difference within the 95% limits of agreement between the pDOI and the rDOI on each sequence (Fig. 4C–E). The mean differences (standard deviation) between the rDOI and pDOI on axial T2WI and axial and fat-suppressed coronal CE-T1WI were 3 ± 3.9 mm, 3.3 ± 4 mm, and 3.4 ± 4.1 mm, respectively. When including measurements from all sequences, nine patients (3.2%) had identical rDOIs and pDOIs, whereas 30 of the remaining 271 patients (10.7%) had rDOIs and pDOIs that differed by more than 10 mm (range, − 20 to 10 mm) (eFigure 1), including 29 patients with an rDOI larger than the pDOI and one patient with an rDOI smaller than the pDOI. Overall, 14 (5%), 11 (3.9%), and 17 (6.1%) of these rDOIs were measured on axial T2WI and axial and fat-suppressed coronal CE-T1WI, respectively.

### Optimal MR measurements for prognostic stratification

Univariate and multivariate analyses using a stepwise Cox regression analysis revealed that age, pNstage, margin status, and extranodal extension, but not sex or betel liquid (both *p* = 0.06), independently predicted OS (eTable 3). Considering the clinical significance of sex and betel liquid, we determined the aforementioned features as confounding factors. Three-knot restricted cubic spline function was determined based on the lowest AIC. The risk of death escalated smoothly with each size increase (eFigure 2). Using an exhaustive algorithm that tested all combinations of different cutoffs, we identified two optimal cutoffs for each MR measurement. These cutoffs and the risk group size, 5-year OS, prognostic differences, and C-index are summarized in eTable 4. In comparisons based on multiple evaluation criteria, with priority given to clinical utility and the C-index, measurements of rDOI on axial CE-T1WI with cutoffs of 8 mm and 12 mm, rDOI measurement on fat-suppressed coronal CE-T1WI with cutoffs of 8 mm and 13 mm, and LD measurement on fat-suppressed coronal CE-T1WI with cutoffs of 14 mm and 20 mm were selected as candidate MR measurements.

**Fig. 5:**
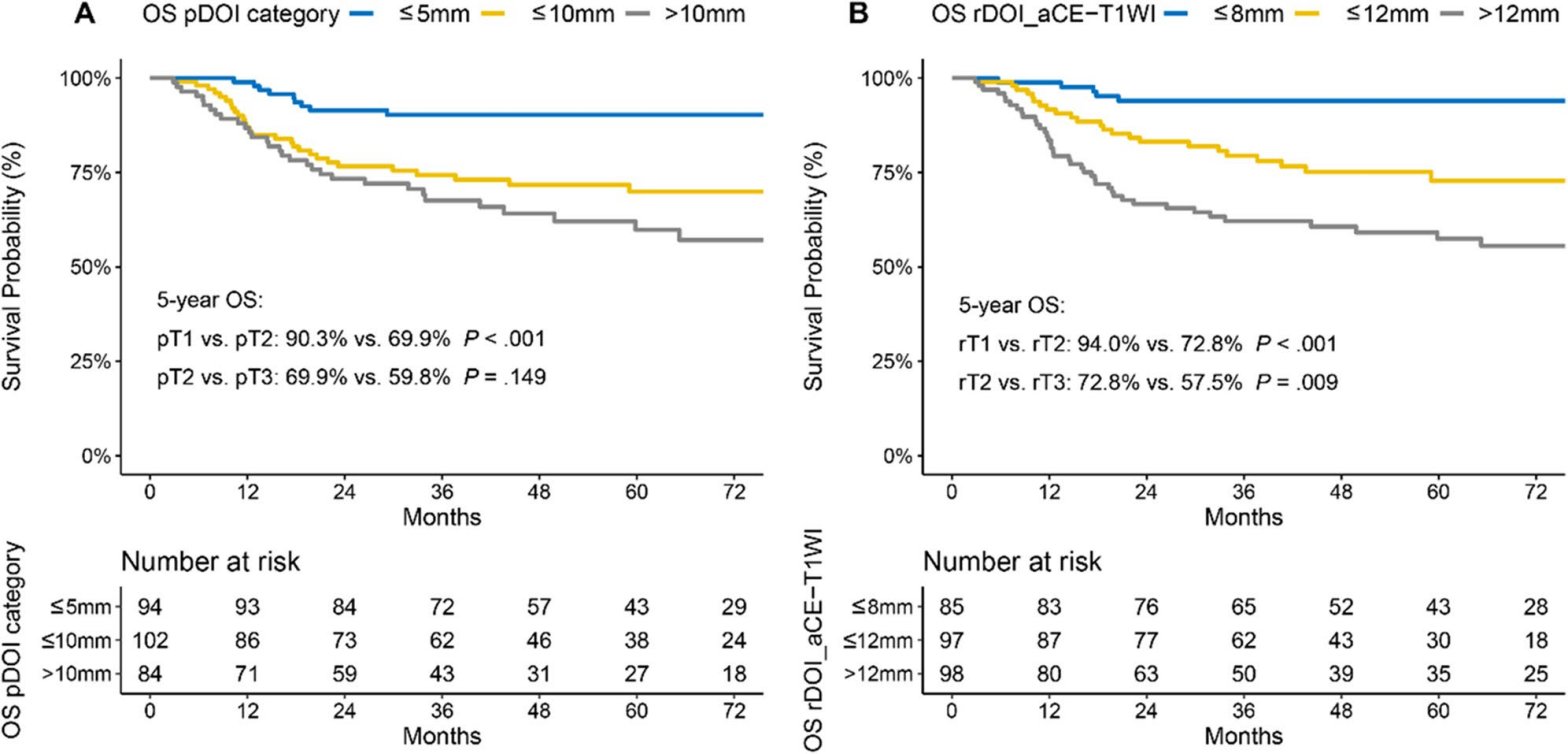
Kaplan–Meier overall survival (OS) curves. Kaplan–Meier curves of OS according to the current pDOI category and proposed optimal MR measurement category in patients with non-pT4 OTSCC. **A** Current pDOI category. **B** Proposed optimal MR measurement category. Abbreviations: OS, overall survival; OTSCC, oral tongue squamous cell carcinoma; pDOI, pathological depth of invasion; rDOI, radiological depth of invasion; aCE-T1WI, axial contrast-enhanced T1WI

The category rDOI measurement on axial CE-T1WI (8 mm and 12 mm as cutoffs) outperformed the other two candidate MR measurement categories and the conventional pDOI category (5 mm and 10 mm as cutoffs) (eTables 4–5), with a larger C-index (0.682 vs. 0.639; *p* < 0.001), better hazard discrimination (0.40 ± 0.36 vs. 0.57 ± 0.38), more balanced sample size (0.38 ± 0.37 vs. 0.48 ± 0.38), greater outcome prediction (29.43 ± 1.45 vs. 27. 6 ± 1.17), and superior 3- and 5-year clinical utility (eFigure 3). However, it presented a slightly inferior hazard consistency (0.56 ± 0.4 vs. 0.44 ± 0.36). The 5-year OS rates for the risk groups according to the proposed optimal MR classification were rT1, 94.0% (as reference); rT2, 72.8% (HR 3.56 [1.35, 9.6]); and rT3, 57.5% (HR 4.33 [1.59, 11.74]), respectively (Fig. 5B, Table 3), whereas those for the 8th edition AJCC risk groups were pT1 90.3%, pT2 69.9%, and pT3 59.8%, respectively (Fig. 5A). The measurement still had a superior impact on OS in both a smaller complete dataset (losing 14.6% of the sample size) without imputation and a larger cohort of patients who received neoadjuvant chemotherapy (eFigure 4).

**Table 3:** Univariate and multivariate analyses of optimal measurement category

Variables	Distribution	5-year	Univariate analysis		Multivariate analysis (enter)	
		OS (%)	HR (95% CI)	*p* value	HR (95% CI)	*p* value
pDOI staging						
≤ 5 mm	94 (33.6%)	90.27	**1 (reference)**	**1 (reference)**	**1 (reference)**	**1 (reference)**
≤ 10 mm	102 (36.4%)	69.94	3.34 (1.58–7.08)	< 0.001	1.84 (0.83–4.09)	0.133
> 10 mm	84 (30%)	59.84	4.82 (2.3–10.1)	< 0.001	2.3 (1.02–5.19)	0.045
rDOI_aCE-T1WI category						
≤ 8 mm	85 (30.4%)	93.97	**1 (reference)**	**1 (reference)**	**1 (reference)**	**1 (reference)**
≤ 12 mm	97 (34.6%)	72.81	4.53 (1.72–11.91)	< 0.001	3.56 (1.35–9.6)	0.011
> 12 mm	98 (35%)	57.48	8.87 (3.5–22.45)	0.009	4.33 (1.59–11.74)	0.004

In Cox regression exploratory analysis with adjustment of age, sex, betel liquid, pNstage, margin status, and extranodal extension (eTable 3), category of rDOI measured on axial CE-T1WI is an independent prognosticator of OS

*pDOI* pathological depth of invasion, *rDOI* radiological depth of invasion, *aCE-T1WI* axial contrast-enhanced T1WI

## Discussion

In this study, the radiological depth of invasion on axial contrast-enhanced T1-weighted imaging showed a prognostic efficacy and clinical utility for OS that was not inferior to that of the pathological depth of invasion, suggesting that it is an appropriate preoperative MR evaluation criterion for primary tumor staging.

MR has been widely used for the preoperative evaluation of patients with OTSCC. False-positive results on MR images and contraction of postoperative histological sections have been reported [7]. The latter, using thresholds of 5 mm and 10 mm, can lead to delayed and disproportionate staging, highlighting the importance of establishing generalizable thresholds on preoperative MR. Some researchers observed an overestimation of rDOI measurement on CE-T1WI due to inflammation or tissue swelling after biopsy and have suggested that the T2 sequence is better for depth measurements. Lam et al [20] chose fat-suppressed T2WI and CE-T1WI on coronal sequences for measuring rTT. They reported that rTT on T2WI was 2 mm greater than pathological sections on average. Mao et al [7] measured rDOI on coronal/axial T2 sequences in 150 OTSCC patients and estimated an average false-positive measurement error of 1.92 mm. In contrast, Yesuratnam et al [8] used T2WI or CE-T1WI either on coronal or on axial sequences, for measurement of rTT in 81 OTSCC patients, with a greater difference between histology and T2WI (3.19 ± 4.87 mm) compared to that between histology and CE-T1WI (2.99 ± 4.41 mm). In our study, compared to other sequences, measurements obtained on T2WI (3 ± 3.9 mm) indeed showed smaller differences; however, this was not our optimal sequence for risk stratification compared to other sequences. We speculate that although there will be an overestimation of the depth for including localized areas with inflammation and swelling after biopsy and this may weaken the correlation of MR-derived measurements with histology, these correspond to peritumoral areas and have a potential impact on survival. This has been verified by Wang et al [21] in their MR study, wherein it was found that radiomics analysis of the additional 10-mm peritumoral extension had an excellent capacity to predict prognosis of OTSCC.

Developing a practical measurement approach that correlates strongly with prognosis rather than pathology may be a priority for guiding staging characterization, surgical planning, and adjuvant treatment management of OTSCC. In this study, the accuracy and reproducibility of measurement depth were affected by comprehensive factors, such as tumor morphology (greater average correlation of 0.79 for exophytic type and smaller average correlation of 0.56 for ulcerative type) [22, 23]. A recent meta-analysis reported that in radiologist-led studies, a pooled, inconsistent, and excessive correlation coefficient between the pDOI and the rDOI/rTT in OTSCC ranged from 0.54 to 0.95 [6]. Another systematic analyses by Lee et al [24] revealed analogical findings. Our measurements were similar to these systematic studies, yielding a slightly inferior and discrepant correlation (*r* = 0.54 to 0.81) in rDOI/rTT. There are several possible reasons for this discrepancy. First, complementary measurements on different sequences may weaken the agreement. Additionally, the influence of measuring depth in dorsal and ventral tumors have rarely been mentioned with the absence of a measurement standard [25].

Second, keeping superficial lesions, linearly enhancing lesions, patchy lesions with minor enhancement, and advanced lesions with deformation of the tongue body, can lead to disagreement as per our measurement experiences [7, 25, 26]. A fat-starved tongue makes measurement more difficult. Third, pseudo-advanced tumors, appearing to be deeply infiltrated on imaging but showing shallow infiltration on pathology and differing by more than 10 mm, exaggerate the measurement disparity [7, 9, 27, 28]. This may partly contribute to the measurement errors for pathologists that do not choose the representative sections, partly due to the shrinkage of tissues [29, 30]. However, the measurement bias is objective and cannot be ignored, ultimately exhibiting inferior and excessive pathology correlation. Furthermore, study design, including sample size, interpretation for measuring depth, and factors mentioned above, such as measurement sequences, dimensions, and intra-observer experience, can impact the final results, making comparisons between studies difficult and implying that an ideal pathology-correlated measurement in OTSCC may not exist.

In the present study, we proposed an MR classification building on previous studies by unifying the sequence of measurements and determining optimal survival-relevant measurement for risk stratification. To address the questions regarding which dimension, tumor size, and sequence are appropriate for preoperative primary staging, we conducted 14 MR measurements, including rTT, rDOI, and LD on routine MR sequences for correlating prognosis, and found that the rDOI on axial CE-T1WI with cutoff values of 8 mm and 12 mm exhibited the best performance in prognostic prediction and risk stratification. In addition, axially unenhanced TIWI had the highest missing rate, making it impossible to obtain complete rDOI measurements. Moreover, the measurable rate for the rDOI was higher on fat-suppressed coronal CE-TIWI than on axial CE-T1WI. However, the predictive efficacy, clinical utility, and sample size balance of the MR-derived measurement were not superior, with minor differences at the second cutoff (13 mm as threshold). Therefore, we recommend measuring rDOI on axial CE-T1WI sequence for OTSCC using thresholds of 8 mm and 12 mm for risk stratification.

Some studies have proposed 8 mm as the cutoff for the rDOI/rTT to predict lymph node metastasis [7, 31], a critical factor in determining patient survival [32]. Similarly, our findings suggest that patients with OTSCC with an rDOI ≥ 8 mm on axial CE-T1WI are at higher risk of mortality. However, unlike previous studies that dichotomized patients into two subgroups, we used two cutoffs to stratify patients into three subgroups. Notably, the overlapping survival curves of patients with pT2 and pT3 disease in the current primary staging system suggest that distinguishing the prognosis of these two subgroups is challenging. Tang et al [33] proposed a second cutoff point of 11.4 mm to predict the pTstage (pT2 vs. pT3). This is similar to our proposal of a second cutoff point (12 mm). To the best of our knowledge, this study is the first large retrospective analysis of OTSCC that compared different measurements of tumor sizes across different MR sequences and proposed two cutoff points that successfully stratify patients into three risk groups by adjusting for detailed clinicopathological variables. The findings provide insight into more appropriate preoperative primary staging for unique oral subsite.

This study had several limitations. First, we used imputation methods in combination with other sequences of the individuals to complete missing sequences. Nevertheless, the proposed measurement categorization still had a superior impact on OS in a smaller complete dataset and in a larger cohort including patients who received neoadjuvant therapy. This will potentially impact the preoperative prognostic stratification and treatment planning in the coming era of neoadjuvant therapy for patients with OTSCC disease. Second, owing to the retrospective nature of our study, we could not control for homogeneity in the MR examinations, which may have affected our measurements. Of note, we did not include DWI in the measurements because multiple factors, including *b*-values, pulse sequences, and field strengths, could influence the stability of the imaging and weaken its measurement reliability. This heterogeneity in MR quality presents a challenge in retrospective rDOI studies. Nevertheless, our proposed MR classification builds on previous studies by harmonizing the sequence of measurements as well as the cutoff values for risk stratification, and provides a prognostic tool that can be used in the real world with imperfect MR examinations. Furthermore, we targeted OTSCC patients with non-pT4 and primarily aimed to recommend an optimal measurement category in clinical staging. However, more radiological factors are warranted for improving preoperative evaluation in survival estimation of whole-staged patients. Finally, to advance research in this area, large multicenter and prospective studies are required to validate our proposed MR measurement.

In conclusion, our study presents an MR primary staging system based on the incorporation of the survival-relevant radiological depth of invasion with thresholds of 8 mm and 12 mm using exhaustive combinations. By stratifying patients into three risk groups with distinct prognoses, MR measurements may provide a more appropriate preoperative primary staging system for quantifying tumor aggressiveness in the unique subsites of the head and neck.

### Supplementary Information

Below is the link to the electronic supplementary material.

Supplementary file1 (PDF 1174 KB)

## Abbreviations

AJCC: American Joint Committee on Cancer
CE: Contrast-enhanced
DOI: Depth of invasion
HR: Hazard ratios
LD: Longest diameter
OS: Overall survival
OTSCC: Oral tongue squamous cell carcinoma
pDOI: Pathological depth of invasion
pTT: Pathological tumor thickness
rDOI: Radiological depth of invasion
rTT: Radiological tumor thickness
TT: Tumor thickness

## Declarations

### Guarantor

The scientific guarantor of this publication is Lizhi Liu.

### Statistics and biometry

Haojiang Li, Hui Xie, and Wenjie Huang kindly provided statistical advice for this manuscript, who are all the co-authors.

### Study subjects or cohorts overlap

Some study subjects or cohorts have not been previously reported.

### Methodology

• retrospective

• diagnostic or prognostic study

• performed at one institution
