# Supplementary material for: Value of radiological depth of invasion in non-pT4 Oral tongue squamous cell carcinoma: implication for preoperative MR T-staging
Source: Eur Radiol. 2024 Feb 3;34(9):6047–59. doi: 10.1007/s00330-024-10598-7 (PMC11364799; doi:10.1007/s00330-024-10598-7)
Supplement: Supplementary file 1 — Supplementary file1 (PDF 1174 KB) [file 330_2024_10598_MOESM1_ESM.pdf]

ONLINE-ONLY SUPPLEMENTS

eTable 1. Measurable rate of each MR measurement

eTable 2. Intra- and inter-observer variabilities for each measurement evaluated in this study (n= 28)

eTable 3. Multivariate analysis for selecting confounding factors

eTable 4. Optimal cutoff points of measurement for rTT, rDOI, and LD in each MR sequence

eTable 5. Performance of category of candidate MR measurement and pDOI in patients with OTSCC

eFigure 1. MR imaging of three cases exhibiting a difference of more than 10 mm between rDOI and pDOI.

eFigure 2. Adjusted hazard ratio (HR) with increasing MR measurement in patients with OTSCC.

eFigure 3. Comparison of the candidate MR measurement and the current pDOI category.

eFigure 4. Kaplan–Meier curves of OS in a smaller completed data set without imputation or a larger data set that involves patients who receive neoadjuvant treatment.

eAppendix. Adjuvant treatment for patients with OTSCC

Supplemental Material

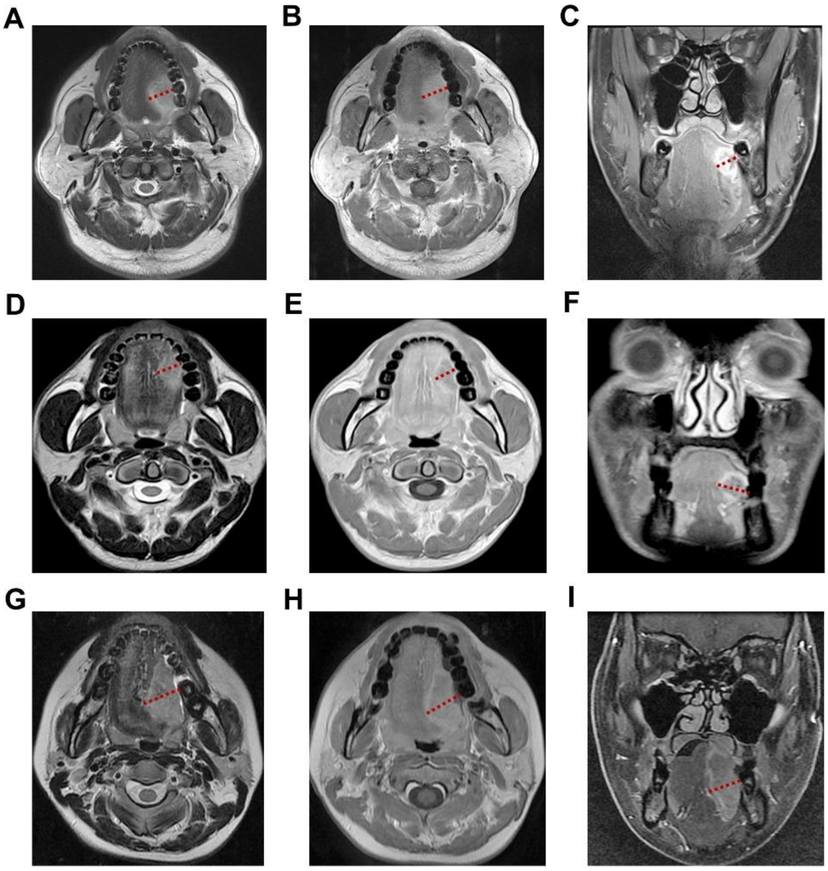

eFigure 1. MR imaging of three cases exhibiting a difference of more than 10 mm between rDOI and pDOI. Case 1: A 41-year-old male with a flat tumor located on the left border, with a pDOI value of 8 mm exhibiting greater rDOI values of 21, 23.2, and 29 mm on axial T2WI (A), axial CE-T1WI (B), and fat-suppressed coronal CE-T1WI (C) images, respectively. Case 2: A 27-year-old man with a flat tumor located on the left border, with a pDOI value of 5 mm exhibiting greater rDOI values of 15.2, 14.6, and 9.8 mm on axial T2WI (D), axial CE-T1WI (C), and fat-suppressed coronal CE-T1WI (E) images, respectively. Case 3: A 33-year-old female with a flat tumor located on the left border, with a pDOI value of 7 mm exhibiting greater rDOI values of 21.5, 21.2, and 22.7 mm on (G) axial T2WI, (H) axial CE-T1WI, and (I) fat-suppressed coronal CE-T1WI images, respectively.

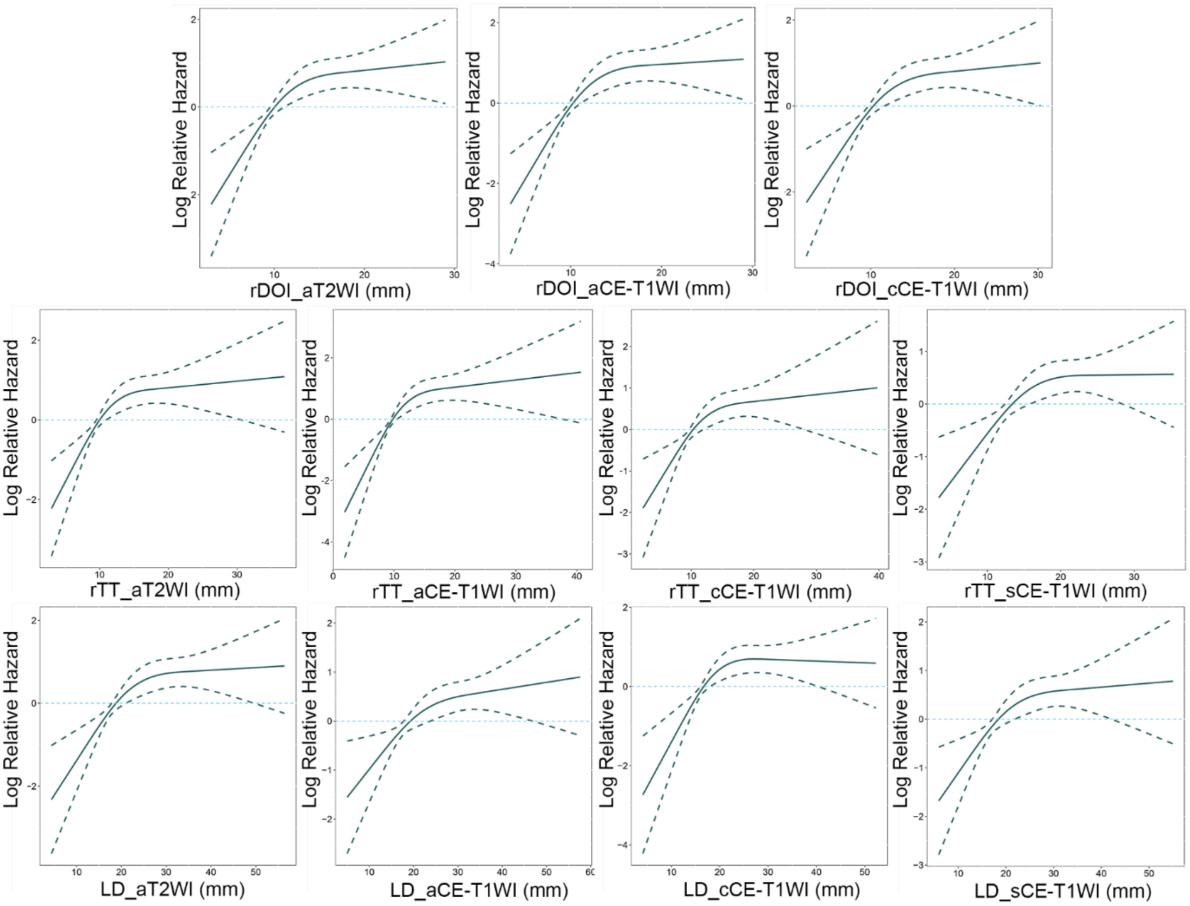

eFigure 2. Adjusted hazard ratio (HR) with increasing MR measurement in patients with OTSCC. Solid lines represent smoothed restricted cubic spline plot of the natural logarithm of predicted adjusted HR versus increasing measurement, and dashed lines represent an estimated 95% CIs of the predicted HRs. The upper, middle, and lower rows represent rDOI, rTT, and LD measurements on each sequence, respectively.

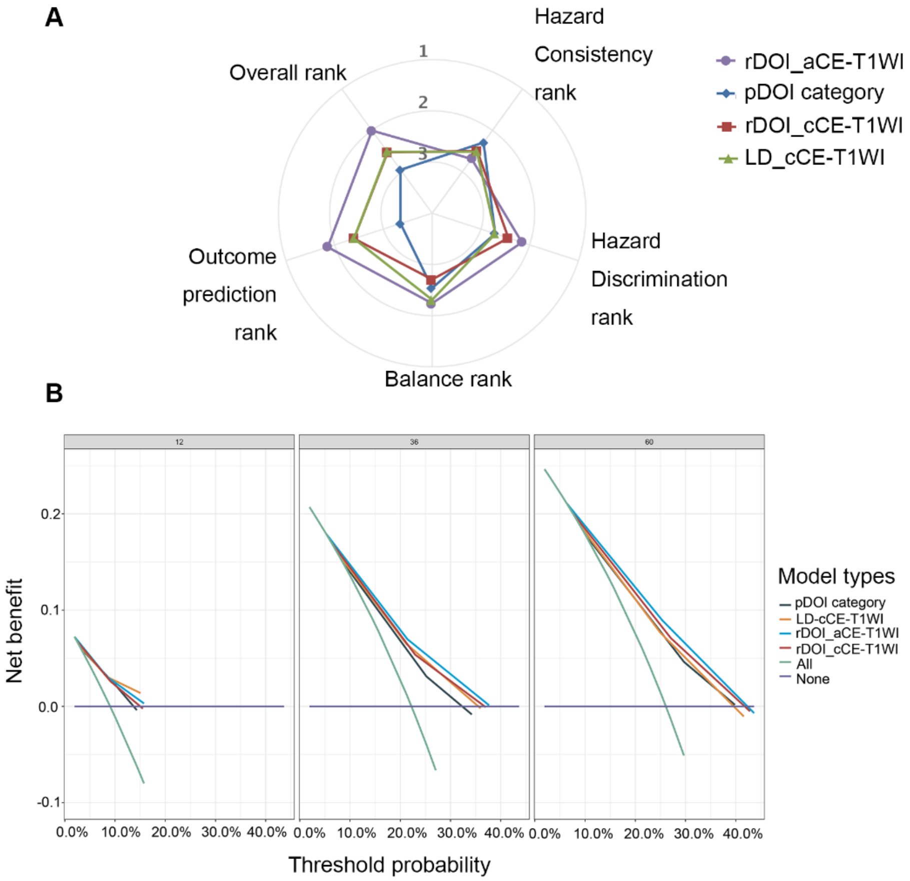

eFigure 3. Comparison of the candidate MR measurement and the current pDOI category. (A) A radar chart for comparison of the five axes representing hazard consistency, hazard discrimination, sample size balance, outcome prediction, and overall rank between optimal MR measurement and pDOI category regarding OS. The closer the rank of hazard discrimination, sample size balance, outcome prediction, and overall rank to 1, the better the corresponding prognosticator. Measurement of rDOI on axial CE-T1WI with cutoff points of 8 mm and 12 mm, having a larger area surrounded by the points on all axes than the pDOI category, representing the superior performance on prognostic stratification. (B) Comparison of 1-, 3-, and 5-year clinical utility between the proposed MR measurement and current pDOI category and other measurement categorization for OS in patients with OTSCC.

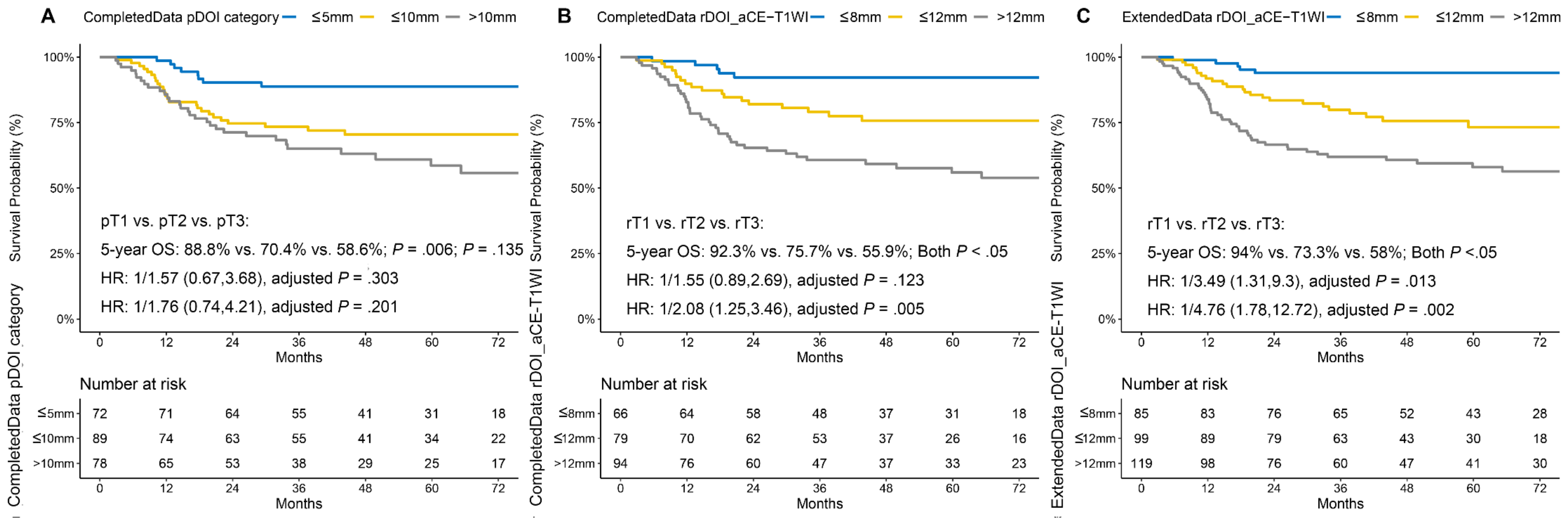

eFigure 4. Kaplan–Meier curves of OS in a smaller completed data set without imputation or a larger data set that involves patients who receive neoadjuvant treatment. (A) Current pDOI category in a complete data set. (B) Measurement of rDOI on axial CE-T1WI in a complete data set. (C) rDOI measurement on axial CE-T1WI in a data set of patients who received neoadjuvant treatment, that is, an extended data set.

eTable 1: Measurable rate of each MR measurement

Variables	Missing rate (%)	Measurable rate (%)
rDOI_aT2WI	4.29	95.71
rDOI_aT1WI	30.3	69.7
rDOI_aCE-T1WI	5.71	94.29
rDOI_cCE-T1WI	3.57	96.43
rTT_aT2WI	1.79	98.21
rTT_aT1WI	23.57	76.43
rTT_aCE-T1WI	3.21	96.79
rTT_cCE-T1WI	3.57	96.43
rTT_sCE-T1WI	5.36	94.64
LD_aT2WI	1.79	98.21
LD_aT1WI	23.57	76.43
LD_aCE-T1WI	3.93	96.07
LD_cCE-T1WI	3.57	96.43
LD_sCE-T1WI	5.36	94.64

Notes. — All measurements on axial unenhanced T1WI have a high missing rate due to difficulty in determining the boundary of lesions. For the measurement of rDOI, coronal fat-suppressed CE-T1WI has the highest measurement rate, and for the measurement of rTT and LD, axial T2WI has the highest measurement rate.

eTable 2: Intra- and inter-observer variabilities for each measurement evaluated in this study (n= 28)

MR measurement		Observer-1	Observer-1[a]	Observer-2	Intra-observer reliability	Inter-observer reliability
**rDOI**	aT2WI	11.4 ± 4.9	14 ± 5.2	12.7 ± 4.3	0.94 (0.88–0.97)	0.9 (0.8–0.95)
	aCE-T1WI	11.6 ± 4.9	13.6 ± 4.9	13.8 ± 5.4	0.95 (0.9–0.98)	0.89 (0.79–0.95)
	cCE-T1WI	11.7 ± 5.2	14 ± 4.5	14.1 ± 5.1	0.81 (0.64–0.91)	0.79 (0.6–0.9)
**rTT**	aT2WI	11.4 ± 5.1	12.8 ± 6.2	14.1 ± 5.6	0.97 (0.94–0.99)	0.96 (0.91–0.98)
	aCE-T1WI	11.4 ± 5	13.5 ± 6.8	13.9 ± 5.3	0.97 (0.93–0.99)	0.87 (0.75–0.94)
	cCE-T1WI	11.7 ± 5.3	14.3 ± 5	14.3 ± 6.9	0.87 (0.74–0.94)	0.7 (0.46–0.85)
	sCE-T1WI	15.4 ± 6.6	14.9 ± 6.6	16.6 ± 4.3	0.79 (0.6–0.9)	0.63 (0.35–0.81)
**LD**	aT2WI	21.4 ± 9	23.4 ± 9.9	27 ± 8	0.89 (0.78–0.95)	0.83 (0.67–0.92)
	aCE-T1WI	21.6 ± 9	24.4 ± 9.8	25.7 ± 7.5	0.95 (0.89–0.98)	0.89 (0.78–0.95)
	cCE-T1WI	19.4 ± 8.1	20.4 ± 8.8	21.7 ± 6.9	0.86 (0.71–0.93)	0.92 (0.84–0.96)
	sCE-T1WI	20.8 ± 8.7	22.2 ± 10.1	22.9 ± 7	0.91 (0.83–0.96)	0.73 (0.5–0.86)

Note. — Intraclass correlation coefficient values are calculated to evaluate the reliability of the intra- and inter-observer agreement on different measurements.

[a]All measurements are performed by the same radiologist after 3 months for testing the reliability of measurement.

eTable 3: Multivariate analysis for selecting confounding factors

Variables[a]	Multivariable cox (Enter)		Multivariable cox (stepwise)	
	HR (95% CI)	*P*-value	HR (95% CI)	*P*-value
**Sex**				
Male	1 (reference)	1 (reference)	1 (reference)	1 (reference)
Female	0.68 (0.33–1.41)	0.303	0.53 (0.28–1.03)	0.060
**Age (y)**				
≤55	1 (reference)	1 (reference)	1 (reference)	1 (reference)
>55	1.76 (1.04–2.97)	0.034	1.66 (1.02–2.72)	0.043
**Smoking**				
No	1 (reference)	1 (reference)		
Yes	1.23 (0.57–2.65)	0.599		
**Alcohol use**				
No	1 (reference)	1 (reference)		
Yes	1.38 (0.63–3.04)	0.426		
**Betel liquid**				
No	1 (reference)	1 (reference)	1 (reference)	1 (reference)
Yes	2.01 (0.75–5.39)	0.167	2.54 (0.97–6.64)	0.057
**cTstage**				
T0	1 (reference)	1 (reference)		
T1	1.25 (0.65–2.39)	0.503		
T2	0.53 (0.15–1.89)	0.326		
T3	1.23 (0.45–3.4)	0.689		
**cNstage**				
N0	1 (reference)	1 (reference)		
N1	0.44 (0.2–0.95)	0.037		
N2	0.81 (0.36–1.82)	0.608		
**pNstage[b]**				
N0	1 (reference)	1 (reference)	1 (reference)	1 (reference)
N1	2.59 (1.11–6.05)	0.027	2.65 (1.25–5.62)	0.011
N2	6.04 (2.98–12.25)	0.000	5.23 (3–9.1)	0.000
N3	0.08 (0–1.49)	0.090	0.14 (0.01–2.11)	0.156
**Perineural invasion**				
No	1 (reference)	1 (reference)		
Yes	1.15 (0.62–2.1)	0.661		
**Margin status**				
Negative	1 (reference)	1 (reference)	1 (reference)	1 (reference)
Positive	8.41 (1.75–40.34)	0.008	8.6 (1.94–38.04)	0.005
**Extranodal extension**				
Negative	1 (reference)	1 (reference)	1 (reference)	1 (reference)
Positive	88.09 (6.18–1255.31)	0.001	38.61 (3.41–437.14)	0.003
**Adjuvant chemotherapy**				
No	1 (reference)	1 (reference)		
Yes	0.64 (0.27–1.5)	0.303		
**Adjuvant radiotherapy**				
No	1 (reference)	1 (reference)		
Yes	1.5 (0.73–3.08)	0.275		

Note. —[a] variables ($P < 0.1$) in univariate analyses (**Table 1**), such as age, betel nut chewing, and margin status, was selected due to their clinical relevance in patients with OTSCC.

[b] Distribution according to pNstage is: pN0, 205 (73.2%) patients; pN1, 24 (8.6%) patients; pN2a, 1

(0.4%) patient; pN2b, 42 (15.0%) patients; pN2c, 1 (0.4%) patient; and pN3b, 7 (2.5%) patients. Owing to pN2a and pN2c being present in only 1 patient each, we combined all subgroups of pN2.

eTable 4: Optimal cutoff points of measurement for rTT, rDOI, and LD in each MR sequence

Variables	n=280	5-Years OS (%)	Univariate analysis		Multivariate analysis (stepwise)			
			HR (95% CI)	*P*-value	HR (95% CI)	*P*-value	C-index	*P*-value
**pDOI category**					Not included[c]		**0.639**	**1 (reference)**
≤5 mm	94 (33.6%)	90.27	1 (reference)	1 (reference)				
≤10 mm	102 (36.4%)	69.94	3.34 (1.58–7.08)	<0.001				
>10 mm	84 (30%)	59.84	4.82 (2.3–10.1)	<0.001				
**AJCC category**[a]							0.636	0.715
≤20 mm	115 (41.1%)	88.89	1 (reference)	1 (reference)	1 (reference)	1 (reference)		
≤40 mm	148 (52.9%)	63.08	3.91 (2.09–7.33)	<0.001	1.99 (1–3.95)	0.049		
>40 mm	17 (6.1%)	66.18	3.52 (1.24–9.99)	0.826	1.27 (0.42–3.88)	0.673		
**LD_aT2WI**							0.665	0.208
≤17 mm	113 (40.4%)	88.7	1 (reference)	1 (reference)	1 (reference)	1 (reference)		
≤25 mm	87 (31.1%)	73.56	2.75 (1.37–5.52)	0.003	1.81 (0.86–3.8)	0.117		
>25 mm	80 (28.6%)	53.32	4.91 (2.54–9.49)	0.029	2.21 (1.05–4.64)	0.036		
**LD_aCE-T1WI**							0.647	0.334
≤16 mm	93 (33.2%)	88.34	1 (reference)	1 (reference)				
≤24 mm	97 (34.6%)	74.98	2.41 (1.14–5.05)	0.017	Not included[§]			
>24 mm	90 (32.1%)	57.92	4.46 (2.21–8.98)	0.02				
**LD_cCE-T1WI**							**0.664**	**0.154**
≤14 mm	84 (30%)	90.85	1 (reference)	1 (reference)	1 (reference)	1 (reference)		
≤20 mm	97 (34.6%)	73.68	3.09 (1.32–7.2)	0.006	2.75 (1.16–6.53)	0.022		
>20 mm	99 (35.4%)	60.13	5.78 (2.59–12.94)	0.015	2.47 (1.01–6.04)	0.048		
**LD_sCE-T1WI**							0.633	0.153
≤15 mm	83 (29.6%)	88.56	1 (reference)	1 (reference)				
≤23 mm	100 (35.7%)	76.38	2.2 (1.02–4.76)	0.038	Not included[c]			
>23 mm	97 (34.6%)	58.85	4.04 (1.95–8.38)	0.019				
**rTT_aT2WI**							0.659	0.13
≤8 mm	93 (33.2%)	89.78	1 (reference)	1 (reference)	1 (reference)	1 (reference)		
≤13 mm	104 (37.1%)	74.58	2.63 (1.22–5.66)	0.011	2.35 (1.08–5.11)	0.032		
>13 mm	83 (29.6%)	55.3	5.42 (2.61–11.25)	0.005	2.32 (1.03–5.25)	0.043		
**rTT_aCE-T1WI**							0.662	0.004
≤8 mm	81 (28.9%)	93.68	1 (reference)	1 (reference)	1 (reference)	1 (reference)		

≤12 mm	105 (37.5%)	72.15	4.44 (1.7–11.56)	<0.001	3.21 (1.21–8.53)	0.019		
>12 mm	94 (33.6%)	59.13	7.8 (3.07–19.82)	0.025	3.77 (1.39–10.27)	0.009		
**rTT_cCE-T1WI**					Not included[c]		0.636	0.131
≤8 mm	83 (29.6%)	88.69	1 (reference)	1 (reference)				
≤12 mm	90 (32.1%)	76.07	2.18 (0.99–4.82)	0.049				
>12 mm	107 (38.2%)	60.54	4.14 (2.01–8.53)	0.019				
**rTT-sCE-T1WI**							0.635	0.221
≤9 mm	58 (20.7%)	94.74	1 (reference)	1 (reference)	1 (reference)	1 (reference)		
≤14 mm	83 (29.6%)	76.96	4.34 (1.28–14.75)	0.01	3.28 (0.96–11.29)	0.059		
>14 mm	139 (49.6%)	63.52	7.77 (2.42–24.95)	0.033	3.97 (1.19–13.25)	0.025		
**rDOI_aT2WI**							0.657	0.154
≤8 mm	93 (33.2%)	89.69	1 (reference)	1 (reference)	1 (reference)	1 (reference)		
≤12 mm	88 (31.4%)	74.48	2.61 (1.19–5.74)	0.014	2.53 (1.14–5.62)	0.022		
>12 mm	99 (35.4%)	58.56	4.98 (2.42–10.27)	0.018	2.25 (1.01–5.02)	0.049		
**rDOI_aCE-T1WI**							**0.682**	**<0.001**
≤8 mm	85 (30.4%)	93.97	1 (reference)	1 (reference)	1 (reference)	1 (reference)		
≤12 mm	97 (34.6%)	72.81	4.53 (1.72–11.91)	<0.001	3.75 (1.41–9.97)	0.008		
>12 mm	98 (35%)	57.48	8.87 (3.5–22.45)	0.009	4.78 (1.78–12.78)	0.002		
**rDOI_cCE-T1WI**							**0.666**	**0.01**
≤8 mm	86 (30.7%)	92.87	1 (reference)	1 (reference)	1 (reference)	1 (reference)		
≤13 mm	106 (37.9%)	71.65	4.1 (1.69–9.92)	<0.001	3.25 (1.32–7.97)	0.01		
>13 mm	88 (31.4%)	58.09	7.26 (3.06–17.23)	0.024	3.78 (1.49–9.56)	0.005		
**rTTmax category**[b]							0.618	0.768
≤9 mm	43 (15.4%)	95.24	1 (reference)	1 (reference)	1 (reference)	1 (reference)		
≤16 mm	121 (43.2%)	75.85	5.1 (1.21–21.47)	0.013	3.5 (0.82–14.91)	0.091		
>16 mm	116 (41.4%)	63.99	8.69 (2.1–35.96)	0.031	3.23 (0.74–14.09)	0.119		
**rDOImax category**[b]							0.657	0.016
≤9 mm	79 (28.2%)	92.34	1 (reference)	1 (reference)	1 (reference)	1 (reference)		
≤13 mm	97 (34.6%)	73.68	3.44 (1.39–8.49)	0.005	3.02 (1.21–7.49)	0.017		
>13 mm	104 (37.1%)	59.67	6.32 (2.68–14.89)	0.021	3.31 (1.33–8.21)	0.01		
**LDmax category**[b]							0.672	0.161
≤18 mm	83 (29.6%)	91.05	1 (reference)	1 (reference)	1 (reference)	1 (reference)		
≤27 mm	101 (36.1%)	75.59	2.8 (1.2–6.57)	0.012	2.12 (0.89–5.05)	0.092		
>27 mm	96 (34.3%)	57.2	6.19 (2.77–13.83)	0.002	2.59 (1.07–6.31)	0.036		

Note. —[a]AJCC category, the largest value of all measurements in each patient and categorized using 20 mm and 40 mm as cutoff points, which mimics the 8th AJCC preoperative T-staging criteria.

[b] rTTmax, rDOImax, and LDmax, the largest value of all rTT, rDOI, and LD measurement in each patient, respectively.

[c] Referring to factors that have been excluded in the multivariate analyses after adjustment of confounding factors.

eTable 5: Performance of candidate MR measurement and pDOI in patients with OTSCC

Evaluation criteria	pDOI	rDOI_aCE-T1WI	rDOI_cCE-T1WI	LD_cCE-T1WI
**Hazard consistency**	5.53 ± 3.12	6.84 ± 4.31	5.95 ± 4.13	5.99 ± 3.74
Score	0.44 ± 0.36	0.56 ± 0.40	0.46 ± 0.39	0.47 ± 0.39
Rank	2.81	3.32	2.95	3
**Hazard discrimination**	0.62 ± 0.14	0.65 ± 0.08	0.64 ± 0.09	0.61 ± 0.10
Score	0.57 ± 0.38	0.40 ± 0.360	0.46 ± 0.38	0.54 ± 0.37
Rank	3.34	2.63	2.92	3.22
**Sample balance**	0.10 ± 0.05	0.09 ± 0.04	0.10 ± 0.05	0.09 ± 0.04
Score	0.48 ± 0.38	0.38 ± 0.37	0.52 ± 0.38	0.41 ± 0.36
Rank	2.96	2.56	3.1	2.71
**Outcome prediction (% variance explained)**	27.6 ± 1.17	29.43 ± 1.45	28.76 ± 1.35	28.71 ± 1.33
Score	80.93 ± 30.33	27.13 ± 34.53	46.82 ± 38.19	48.05 ± 37.32
Rank	4.11	2.11	2.76	2.89
**Overall score**	2.29 ± 0.69	1.61 ± 0.72	1.90 ± 0.75	1.91 ± 0.72
Overall rank	3.6	2.38	2.92	2.96
% Rank=1	88	336	221	188

Note. —The four evaluation criteria defined by Groome et al. [19] for evaluating staging category include hazard consistency, hazard discrimination, sample size balance, and outcome prediction. The lower scores of hazard consistency, hazard discrimination, and sample size balance and higher scores of outcome prediction indicate better performance.

Adjuvant treatment

Patients with adverse pathological characteristics, including pT3/4, pN2/3, extranodal extension, positive margin, perineural and lymphovascular involvement, and lower (level IV or V) neck involvement, were recommended adjuvant radiotherapy or adjuvant chemotherapy under the National Comprehensive Cancer Network guidelines [1]. A total of 49 of 280 (17.5%) patients received cisplatin-based chemotherapy consisting of 60 mg/m^2 cisplatin plus 260 mg/m^2 paclitaxel weekly or 80–100 mg/m^2 cisplatin on days 1, 22, and 43 of radiotherapy. Target volumes were delineated slice by slice on the treatment-planning CT scans using an individualized delineation protocol following the International Commission on Radiation Units and Measurements reports 62 and 83. The gross tumor volumes (GTVs), including the primary tumor (GTVnx) and involved cervical lymph nodes (GTVnd), were determined from the imaging findings at presentation.

The clinical target volume in the high-risk regions (CTV-1) included the GTVnx with a 5–15 mm margin (if possible) and the whole superficial oral tongue [2]. The CTV for the low-risk regions (CTV-2) covered all at-risk uninvolved nodal levels, which varied according to the site and laterality of the primary tumor, as well as the extent of neck dissection (e.g., whether a contralateral neck dissection was performed for a midline tumor). The planning target volumes (PTVs) were determined from the GTVs or CTVs, with a 3–5 mm margin for setup variations. The radiation doses for each target volume were as follows: 66–72 Gy in 30–33 fractions to the PTV of GTVnx; 64–70 Gy to the PTV of GTVnd; 60–66 Gy to the PTV of CTV-1; and 54–62 Gy to the PTV of CTV-2. Salvage treatment for recurrent and refractory cases included surgery, reirradiation, and chemotherapy.

ONLINE-ONLY REFERENCES

1. National Comprehensive Cancer Network (2023) NCCN Clinical Practice Guidelines in Oncology-Head and Neck Cancers. Pennsylvania, PA: NCCN.

2. Evans M, Beasley M (2018) Target delineation for postoperative treatment of head and neck cancer. Oral Oncol 86:288–295.
